# MELK—a conserved kinase: functions, signaling, cancer, and controversy

**DOI:** 10.1186/s40169-014-0045-y

**Published:** 2015-03-07

**Authors:** Ranjit Ganguly, Ahmed Mohyeldin, Jordyn Thiel, Harley I Kornblum, Monique Beullens, Ichiro Nakano

**Affiliations:** Department of Neurological Surgery, The Ohio State University, 385 Wiseman Hall, 410 W 12th St., 43210 Columbus, OH USA; The James Comprehensive Cancer Center, The Ohio State University, Columbus, USA; Semel Institute for Psychiatry and Human Behavior and the Jonsson Comprehensive Cancer Center, UCLA School of Medicine, Columbus, USA; Laboratory of Biosignaling & Therapeutics Department of Cellular and Molecular Medicine Campus Gasthuisberg, Gasthuisberg, Belgium

**Keywords:** Maternal embryonic leucine zipper kinase, MELK, Cancer, Development, Glioblastoma, Glioma-initiating cells, Cancer-initiating cells, Mitotic kinase, Cancer therapeutics

## Abstract

Maternal embryonic leucine zipper kinase (MELK) is a highly conserved serine/threonine kinase initially found to be expressed in a wide range of early embryonic cellular stages, and as a result has been implicated in embryogenesis and cell cycle control. Recent evidence has identified a broader spectrum of tissue expression pattern for this kinase than previously appreciated. MELK is expressed in several human cancers and stem cell populations. Unique spatial and temporal patterns of expression within these tissues suggest that MELK plays a prominent role in cell cycle control, cell proliferation, apoptosis, cell migration, cell renewal, embryogenesis, oncogenesis, and cancer treatment resistance and recurrence. These findings have important implications for our understanding of development, disease, and cancer therapeutics. Furthermore understanding MELK signaling may elucidate an added dimension of stem cell control.

## Introduction

As a member of the AMPK/Snf1 family, Maternal Embryonic Leucine-zipper Kinase (MELK) encodes a serine/threonine kinase that is highly conserved across a variety of mammalian and non-mammalian species. The MELK gene was initially cloned from mice (*Mus musculus*) using differential display analysis of cDNA libraries from unfertilized eggs and preimplantation embryos [1]. This experiment identified MELK to be one of three uniquely stored maternal mRNAs expressed in the developing egg and embryo [1]. Due to the presence of a conserved serine/threonine kinase domain in the N-terminal region, MELK was classified as a novel member of the AMPK/Snf1 family [1,2]. Similar to the other family members, the catalytic domain of MELK is followed by a Ubiquitin-Associated (UBA) domain, which is essential for its catalytic activity [3,4]. The kinase activity of the other AMPK/Snf1 family members is dependent on phosphorylation in their activation loop by upstream kinases (such as LKB1 or CaMKK2). Interestingly, MELK is activated by auto-phoshorylation *in vitro* [5]: a unique mechanism among the AMPK/Snf1 family members.

Shortly after the discovery of MELK in mouse egg and preimplanation embryos, a second group cloned MELK—also known as MPK38 (Murine protein serine/threonine kinase 38)—from a murine teratocarcinoma cell line, PCC4 [6]. The group went on to show a wide expression pattern in adult tissues, and found that MELK is expressed in the thymus and spleen, but not present in muscle, kidney, or liver. Interestingly, MELK expression was restricted to T lineage cells and macrophage/monocyte cells, but was not detectable in a B cell line [6]. MELK was found to have active kinase catalytic activity in an immune complex kinase assay, suggesting a functional gene product was indeed formed in these tissues [6]. The authors postulated that MELK plays an important role in signal transduction of certain lineages of hematopoietic cells.

Since those seminal discoveries, more groups have examined the expression patterns of MELK using different organ systems and cell types, and across different species in both normal and neoplastic cells. As a result, we have a better understanding of some basic mechanisms, functions, and signaling pathways involving MELK, including interactions that link it with tumor progression. Though these studies have clarified basic functions, additional studies are required to ascertain specific roles and pathways. Knockdown studies, using short hairpin RNAs (shRNA) and small molecule inhibitors, and forced overexpression studies have allowed for careful experiments to unravel the precise cellular functions of MELK. These studies, discussed in subsequent sections, have implicated MELK in a number of cellular processes, and suggest an important role for MELK in cancer biology.

**Figure 1:**
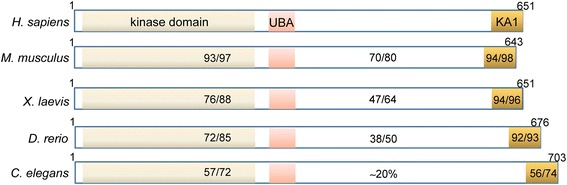
**The domains essential for the kinase activity, namely the kinase, UBA and KA1 domain are highlighted.** The numbers indicate the % of identical/conserved residues in the different domains (kinase + UBA domain residues 11-326, variable domain 327-600, KA1 residues 601-651 in human MELK) for the different species compared to human Melk (H. sapiens) as revealed by a Blast search.

The identification of the protein structure of human MELK has enabled investigators to study MELK orthologs in various species. The protein structure of MELK has been mainly conserved across various mammalian and non-mammalian species as depicted in Figure 1. Interestingly, the functional roles of MELK appear to be slightly different in each species. MELK orthologues in *Xenopus laevis* have been shown to interact and phosphorylate key proteins to regulate G2/M cell cycle progression [7]. As a result, MELK has been strongly postulated to play a functional roll in cell cycle regulation, proliferation, mitosis and spliceosome assembly [8-11].

In non-mammalian systems such as *Caenorhabditis elegans*, *Danio rerio* (zebra fish), and *Xenopus*, MELK plays an important role in cell division, as well as propagation and maintenance of some organ-specific stem cells [12-14]. Likewise, in mammalian systems, MELK is essential for organogenesis, stem cell proliferation, and cell cycle regulation. Interestingly, MELK is additionally involved in the development of numerous human cancers, tumor initiation, and tumor propagation [15-17]. Although recent studies have shed light on the context-dependent, diverse physiological roles of MELK and its involvement in various signaling pathways, MELK’s functions and regulatory mechanisms are just starting to be understood.

## Review

### Cell cycle regulation: Pro-apoptotic and anti-apoptotic functions of MELK

MELK’s expression patterns in mammalian and non-mammalian systems, and its associations with cellular proteins, cued scientists to explore its role in cell cycle regulation. For example, MELK expression is restricted to proliferating cells in early embryonic development, and is expressed at particularly high levels in cancer cells [10]. In addition, in the TC7 intestinal cancer cell line, MELK expression levels cycle similarly to those of cyclin A, cyclin B, and CDK4 [10]. In brain tumors, MELK mRNA expression is co-regulated with that of known mitosis-phase regulatory proteins such as ASPM and Aurora Kinase B [18]. Thus the regulation and expression levels of MELK correlate with other kinases and enzymes involved in cell cycle progression. These data underscore a a prominent role for MELK regulating cell cycle progression. In addition, depletion of MELK with siRNA caused a G1/S phase cell cycle arrest in GBM [17], suggesting MELK may even be required for cell cycle progression. Notably, similar conclusions can be drawn from data collected from nonmammalian systems. For example, in *Xenopus,* the cycling of MELK depends on its phosphorylation during M-phase, stabilizing it, whereas dephosphorylation of MELK coincides with its degradation [10].

It is important to note that MELK’s effect on promoting or inhibiting apoptosis is still an area of active research, as evidence for both of these roles exists. Jung et. al. showed in mice, that MELK phosphorylates Apoptosis Signal-regulating Kinase 1 (ASK1). This induces H_2_O_2_-mediated apoptosis in embryonic kidney and hematopoietic cells [19]. In (HCT116) colon cancer cells, MELK interacts with p53, and overexpression of MELK increases p53 expression proportionately in vitro and in vivo [20]. MELK also phosphorylates the Ser15 residue on p53 and stimulates its activity. Importantly, this pro-apoptotic function of MELK is also observed in some non-mammalian systems. For example, MELK has a role in promoting apoptosis in *C. elegans* through a caspase-independent cell extrusion method (described later) [21]. Together, these studies suggest MELK may have a critical role in promoting apoptosis in developmental models and some forms of cancer.

In contrast, data from other groups suggest MELK is anti-apoptotic. In glioblastoma (GBM) cells, a highly malignant brain cancer, the expression of p53 exhibited an inverse correlation with MELK expression. MELK silencing increased p53 expression and induced p53-dependent apoptosis [22]. In HEK 293T cells, the promoter activity of p53 diminished with MELK overexpression, whereas knockdown of MELK with shRNA yielded the opposite result. Functionally, p53 inhibition partially rescued MELK depletion-mediated GBM cell apoptosis. Given that MELK and p53 appear to be exclusively expressed in glioma cells, we proposed that MELK negatively regulates p53 activity and vise versa. We postulate that MELK has anti-apoptotic actions through its regulation on p53. In breast cancer models Lin et al. showed that the effect of MELK on cancer cell growth is associated with resistance to apoptosis through the inhibition of a pro-apoptotic function of Bcl-G [23], although these results are challenged by other studies [24].

It is possible that MELK may be involved in both pro- and anti-apoptotic pathways indicating a possible context-dependent function. Evidence for this possibility is the paradoxical involvement of MELK in promoting cell division and also cell death in C. elegans [12,21]. Overall, these studies support dual roles for MELK and continued research will give us better understanding of the regulators and biological contexts in which MELK is pro-apoptotic and anti-apoptotic.

### MELK in mammalian organ development and tissue homeostasis

Members of the AMPK/Snf1 family in the mammalian system are generally associated with cell survival under conditions of nutrient starvation [25]. MELK appears to regulate mitotic cell progression in early embryogenesis [1], in somatic stem cells [15], and in certain cell lineages of the hematopoietic system [6].

MELK is preferentially expressed by immature cells in the brain including neural stem cells (NSCs) throughout mouse development [9]. Furthermore, NSCs depend on MELK for their proliferation, thus implicating MELK in mitotic progression and stem cell maintenance. In the mouse central nervous system, MELK expression is restricted to early and mid-embryonic developmental stages and is absent in the adult brain [9]. In particular, MELK mRNA is strongly expressed within periventricular germinal zones, but is largely absent outside the germinal zones suggesting that post-mitotic differentiated cells lack MELK expression. This expression pattern is similar to that observed with zMELK (zebrafish ortholog) [13], supporting a conserved role for MELK in maintaining stem/progenitor identity. MELK is also expressed in the mouse postnatal hippocampus, which is the other region where neural stem cells reside [9]. Neurosphere cultures derived from embryonic and postnatal subventricular zone tissues demonstrate that forced MELK overexpression increased the number of self-renewing neurosphere-forming cells, whereas knockdown showed significant reduction. Collectively, in the mouse brain, MELK is expressed by neural stem cells and regulates their proliferation.

### MELK in cancers and cancer stem cells

Preferential upregulation of MELK in cancers and recent data from knockdown and forced overexpression studies suggest that MELK promotes cancer cell growth. MELK overexpression has been identified in several human cancers: prostate [26], breast [27], brain [28], colorectal [29] and gastric [8]. In glioblastoma (GBM), mRNA and protein expression of MELK is highly unregulated, and inversely correlates to patient post-surgical survival [28]. Increased MELK expression was detected in particularly aggressive subtypes of breast cancer such as basal-like breast cancer (BBC), and correlates with poor prognosis [27,30]. Knockdown of MELK with shRNA (breast cancer cell line T-47D) resulted in decreased proliferation of cells both in vitro and in vivo [27]. MELK knockdown decreased proliferation and anchorage-independent growth in vitro, and decreased tumor growth in vivo in breast, pancreatic, and colorectal carcinomas [29]. These findings suggest that MELK activity is implicated in tumor growth and aggressiveness, and inhibition of MELK may be an attractive cancer therapeutic target.

The isolation and characterization of cancer stem cells (CSCs) from several types of primary cancers has provided a major paradigm shift in our understanding of cancer biology [31]. The cancer stem cell hypothesis postulates that tumor cells are hierarchically organized with respect to tumor growth initiation and treatment resistance. Furthermore, cancer stem cells represent a subpopulation of cancer cells with tumor-initiating capability. These cells give rise to a variety of tumor cells in response to cellular and environmental signals, and are hypothesized to play a role in tumor initiation and propagation [32]. Cancer stem cells have become a novel and attractive therapeutic target because of the pivotal role they play in treatment, resistance, and recurrence, as they are a source of cell renewal within the tumor.

MELK has been shown to be differentially expressed in cancer stem cells [16]. MELK knockdown by small interfering RNA (siRNA) or short hairpin RNA (shRNA) induced apoptosis of cancer stem cells in GBM stem cells (GSCs) both in vitro and in vivo. In contrast, MELK knockdown arrested proliferation of mouse neural precursors without causing a significant increase in cell death, at least in vitro. Of note, MELK silencing in vivo induced glial differentiation of GSCs resulting in less aggressive, lower grade tumors. While MELK regulates NSC and GSC proliferation, it likely only plays a role in GSC survival and not NSC survival. This key distinction makes MELK an attractive therapeutic target in GBM.

In colorectal carcinoma, MELK expression was elevated when compared to normal tissues, and was found to be spontaneously elevated in a murine model of intestinal tumorigenesis [29]. MELK also localized to tumor cells and in particular the basal regions of crypts of normal gastrointestinal epithelium—the stem cell location in normal colonic tissue [29]. This finding suggests MELK overexpression may be involved in the malignant transformation of normal stem cells. In addition, MELK is preferentially expressed in proliferating cells in normal mammary gland luminal cells [33]. These cells are thought to be the origin of mammary ductal tumors in humans and mice [34]. This underscores the possibility that MELK upregulation may be involved in tumor formation. Conclusions derived from these studies suggest that MELK provides a unique growth advantage in cancer, and that it may be doing so by playing an important in maintaining the properties of the cancer stem cell population.

MELK may also play an interesting role in tumor resistance to therapies. In GBM and other High Grade Gliomas (HGG), experimental radiation treatment strongly upregulated MELK in vitro and in vivo [22,35]. This indicates that MELK is likely a stress-induced kinase. In addition, blocking MELK upregulation in radiation-treated glioma stem cells (GSCs) with shRNA-mediated silencing, and inhibition of MELK with a small molecule kinase inhibitor C1 resulted in increased cellular apoptosis. However, MELK overexpression promoted cancer cell growth [36]. Similar findings were observed in models of colorectal cancer [37]. Treatment of the rectal cancer cell line SNU-503 with 5-flourouracil or radiation increased MELK expression [37]. In addition, knockdown of MELK with siRNA decreased cell proliferation and caused changes in the cell cycle after radiation or 5-FU treatment. These pharmacological data indicate that upregulation of MELK contributes to the survival of tumor cells, particularly when they suffer from radiation insult. Because the inhibition of MELK has a greater effect on cancer stem cells than normal stem cells, the development of novel pharmaceutical therapies that target MELK is an active area of clinical investigation.

### Signaling mechanisms that mediate MELK action

**Figure 2:**
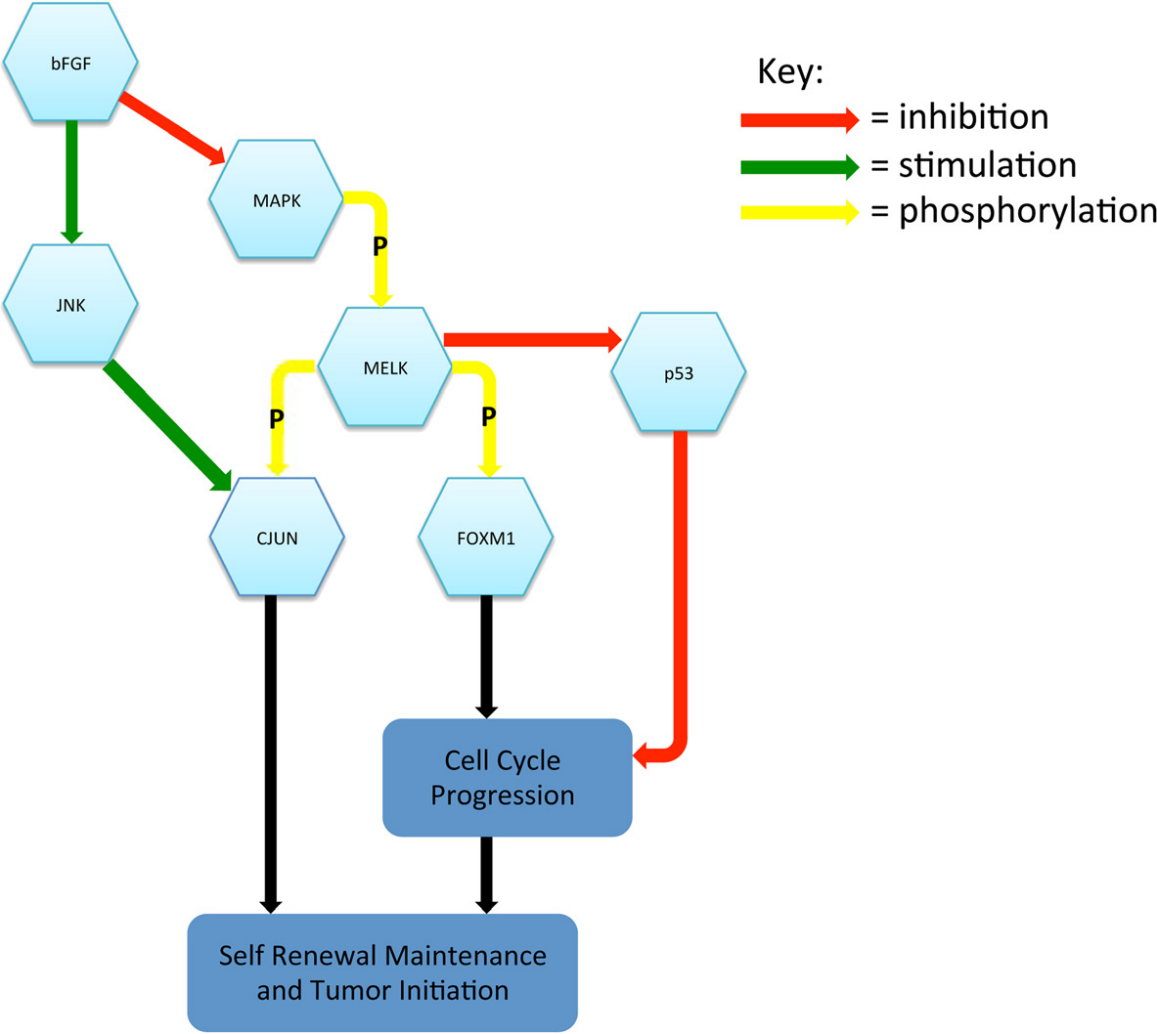
**This schematic highlights many of the signaling pathways MELK is involved in.** Upregulation of MELK drives cell cycle progression and tumor formation. Since many mysteries still surround MELK, more factors will likely be discovered to play a role in MELK regulation and signaling pathways.

Since the discovery of MELK, many signaling proteins and pathways that regulate the action of MELK have been discovered, some of which are highlighted in Figure 2. Early biochemical analyses found that exogenously expressed murine MELK binds to the zinc-finger-like Zpr9, which results in the activation of the oncogenic transcription factor B-Myb in murine cell lines [38].

MELK both regulates and is regulated by one family of MAP kinases, the c-Jun NH(2)-terminal kinases (JNKs) in a cancer-specific manner [22]. The JNK pathway is required for the regulation of cell proliferation, apoptosis, and inflammatory responses in cancers, including HGGs. Data indicate that MELK binds c-JUN, the downstream oncogenic transcription factor target of JNK, in GSCs, but not in normal progenitors. This binding of c-JUN to MELK is diminished when the kinase activity of MELK is nullified, suggesting that the kinase activity of MELK is required for its interaction with c-JUN. Thus, the tumor-specific MELK interaction with JNK/c-JUN is likely one of the mechanisms for the selective apoptosis that occurs as a result of MELK inhibition in GSCs, but not in normal progenitors.

In cancer cells, MELK forms a protein complex with the transcription factor/oncogene FOXM1, a master regulator for cell cycle progression [35]. FOXM1 is overexpressed in a number of human cancers including GBM. MELK-regulated phosphorylation of FOXM1 facilitates FOXM1 transcriptional activity and induces the expression of various mitotic regulators (e.g. Survivin, Aurora B, and CDC25B). Based on the evidence that FOXM1 directly interacts and is phosphorylated by MELK in GSCs, MELK may orchestrate the priming event of the complex signaling toward p53, VEGF, and Wnt/β-catenin in cancers including GBM.

### A target for therapy

Given the preferential upregulation of MELK in cancers and recent experimental data suggesting the positive role of MELK on cancer cell growth, MELK has been recognized as a potential therapeutic target for brain, colorectal, lung, and ovarian cancers [29]. In light of these preclinical studies, novel therapeutics have been developed to selectively target MELK in cancers. As demonstrated by Compound 1 (C1) and OTSSP167, discoveries of novel Small Molecule Drugs (SMDs) that inhibit MELK can be potential therapies for cancers with high levels of MELK expression [36,39,40]. For example, OTSSP167 suppressed mammosphere formation of breast cancer cells, and suppressed tumor growth in xenograft studies of breast, lung, prostate, and pancreas cancer cell lines in mice by both intravenous and oral administration [41]. Currently, OTSSP167 is in a Phase I, single-center, cohort dose escalation trial for patients with any locally advanced or metastatic solid tumor malignancies refractory to current treatments (clinicaltrials.gov identifier: NCT01910545). This study, being conducted by OncoTherapy Science Inc. at the University of Chicago since August 2013, is the first human trial of a MELK inhibitor. The final data collection for primary outcomes is estimated to occur by December 2015, the results of which are highly anticipated.

### Role of MELK in neuroblast divisions in *C. elegans*

Much of our understanding of the functional roles, signaling mechanisms, and structure of MELK comes from findings in nonmammalian species. Orthologs of MELK have been identified in a few species, and allow us to study the role of MELK in developmental biology. PIG-1 is the MELK orthologue in *C. elegans.* As depicted in Figure 1, a high degree of homology exists between the N-terminal kinase domain (72%) and the C-terminal KA1 domain (74%) of PIG-1 and mouse Melk, while the remaining domains are much less conserved.

Physiologically, PIG-1 has been implicated in the regulation of asymmetric neuroblast divisions involving neural lineages that divide during embryogenesis until the first larval stage [12]. These neuroblast divisions generate one smaller, anterior offspring fated to undergo apoptosis, and one larger, posterior offspring fated to become a neural precursor. Mutations of the gm280, gm300, and gm301 alleles of PIG-1 result in a higher penetrance of neurons due to an abnormal, centrally-located mitotic spindle. This mutation yields two neuronal precursors rather than one, resulting in a subsequent increase in the number of neuroblast cells. PIG-1 is localized to centrosomes in dividing neuroblasts independently of its kinase activity, but the function of this centrosomal localized PIG-1 remains unknown [42]. Mechanistically, the regulation of asymmetric neuroblast divisions by PIG-1 is controlled by the phosphorylation of the conserved Thr169 in its activation loop by PAR-4 (the LKB1 homolog in *C. elegans*). This suggests that PIG-1/MELK may be regulated by upstream kinases *in vivo*. Thus, PIG-1 in *C. elegans* may contribute to determine the daughter cell fates in neuroblasts.

In contrast to these studies describing the positive roles of PIG-1 in cell division, Denning et. al [21] demonstrated that PIG-1 may also promote cell death through a caspase-independent cell extrusion mechanism. The proposed role for PIG-1 is the prevention of cell surface expression of cell-adhesion molecules. This results in detachment and subsequent cell death of the shed cell. Remarkably, this process is also dependent on the PAR-4 kinase complex, indicating the significance of the PAR-4/PIG-1 signaling axis in *C. elegans*.

### MELK in zebra fish hematopoiesis

A MELK-like gene (zMELK) was identified in zebra fish (*Danio rerio*) based upon high homology of the kinase, UBA, and KA1 domain with mouse and human MELK [13]. Additionaly, zMELK and human MELK genes are preferentially expressed in embryonic brains and retinas, especially in the proliferative ventricular zones, where neural stem cells are localized. In the adult zebra fish however, zMELK expression is limited to a subset of hematopoietic tissues [43]. zMELK likely plays an essential role in organogenesis, as disrupted zMELK expression by Morpholino injection resulted in numerous developmental abnormalities including severe anemia, retarded eye development, a swollen cerebral tectum, and a slower heart rate. The exact mechanisms underlying individual abnormalities largely remain undetermined. The development of severe anemia induced by Morpholino injection suggests that zMELK is essential for primitive hematopoiesis. Nonetheless, the overall phenotype of zMELK knockdown in zebra fish embryos is rather subtle, unlike the results by introducing MELK mutation in *Xenopus* and *C. elegans*. These phenotypic differences may arise from structural, regulatory, or signaling differences of zMELK compared to other MELK orthologs.

### MELK in *Xenopus laevis* embryogenesis

**Figure 3:**
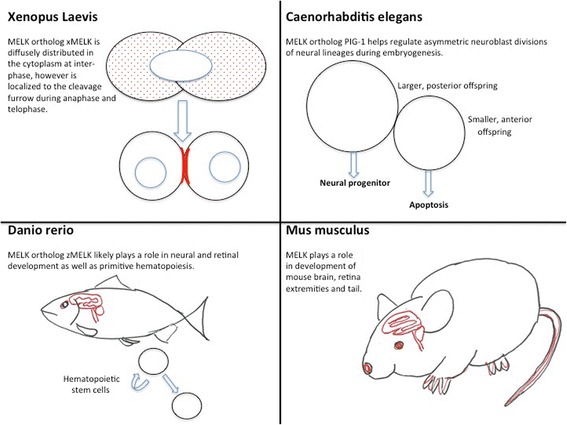
**This schematic illustrates the various developmental functions of MELK.** MELK expression is represented by red coloring. Blue represents a nuclear envelope.

In *Xenopus laevis*, the MELK ortholog pEg3 or xMELK shows the same domain structure as human MELK (Figure 1) except for the lack of the leucine zipper as in zMELK. xMELK is critical for proper cell division, and furthermore, altering xMELK expression either by knockdown or overexpression leads to abortive cell divisions in Xenopus embryos. This indicates that tight control of xMELK expression during the cell cycle is critical [14,44]. As the cell prepares to enter mitosis, xMELK protein is diffusely distributed in the cytoplasm at interphase, however, during anaphase and telophase, xMELK is associated with the cleavage furrow of dividing cells. This pattern of expression indicates MELK is likely involved with proper completion of cell division [14]. The distribution of MELK in the cell during these phases of the cell cycle are displayed in Figure 3.

Overall, non-mammalian MELK has several unique functions in individual organisms mostly related to cell division and daughter cell fates. MELK regulates asymmetric cell division and apoptotic cell death in *C. elegans*, hematopoiesis in zebra fish, and cytokinesis in *Xenopus*. Though the theme of cell division is common among the MELK orthologs, each has a unique function. These differences may be due to slight variations of sequence and domains, organ-specific expression patterns, and molecular interactions. Further studies are needed to understand the specific functions of MELK in each system.

## Conclusion

In a variety of systems and organisms, MELK appears to be highly conserved with a significant effect on proliferation, cell cycle regulation, and apoptosis. Aberrant regulation and activity of MELK is implicated in a variety of human cancers predominantly because of these properties, but also possibly because of its effect on cancer stem cells. In addition, inhibiting MELK may have potential therapeutic value as preliminary studies with the use of knockdown strategies and small molecule inhibitors have revealed promising results in vitro and in vivo. Although there appears to be a broad spectrum of functions for MELK, detailed mechanisms with upstream and downstream signaling pathways still need to be identified. In addition, several lines of research demonstrate that MELK may have both pro-apoptotic and anti-apoptotic effects. This dual functionality appears to be dependent on biological context and the system being studied, and more research in this area is required to identify these variables. Various nonmammalian species represent important systems in which to study these variables. Additional studies will have an exciting impact on our understanding of MELK and its effect on development and disease.
